# Awareness, Knowledge, and Uptake of the Pertussis Vaccine During Pregnancy and Postpartum: A Cross-Sectional Study

**DOI:** 10.7759/cureus.106986

**Published:** 2026-04-13

**Authors:** Mada Alsharif, Ibrahim Noor Elahi

**Affiliations:** 1 Preventive Medicine, Ministry of Health, Jeddah, SAU; 2 Preventive Medicine and Public Health, General Medical Commission, Ministry of Health, Jeddah, SAU

**Keywords:** awareness, knowledge, maternal pertussis vaccine, mpv, tdap

## Abstract

Background and aim

Maternal pertussis vaccination (MPV) with tetanus, diphtheria, and acellular pertussis during pregnancy is an effective strategy to protect young infants from severe pertussis infection. Despite national and international recommendations, uptake of MPV remains suboptimal in many settings, including Saudi Arabia. The aim of this study is to assess awareness, knowledge, uptake, and intention to receive MPV among pregnant and recently postpartum women and to identify factors associated with it.

Methods

A cross-sectional study was conducted among pregnant women in their third trimester and women within 30 days postpartum attending the obstetrics and gynecology clinic at King Abdullah Medical Complex, Jeddah. A validated, self-administered questionnaire adapted from previous research was used for data collection. The calculated sample size was 338, and participants were selected using simple random sampling. Eligible women were approached by the primary researcher in the outpatient clinic and maternity ward.

Results

Among 351 participants, MPV uptake was very low, with only 10 women (2.8%) vaccinated. Nevertheless, 109 participants (31.1%) reported an intention to receive MPV if it were offered free of charge. Awareness and knowledge levels were mostly low among study participants. In the univariate analysis, higher knowledge levels were associated with MPV uptake (crude OR = 6.619; 95% CI: 1.814-24.151). In contrast, lack of or uncertainty regarding physician recommendation was associated with lower uptake (crude OR = 0.971; 95% CI: 0.953-0.989).

Conclusions

MPV uptake of less than 3% in this study reveals a substantial gap between national policy and clinical practice, consistent with findings from comparable settings regionally and internationally. These findings highlight clear and actionable priorities: routine MPV counseling should be integrated into antenatal care protocols, vaccine accessibility should be improved, awareness campaigns should be strengthened through media channels, and the implementation mechanisms of the national program should be reevaluated. In addition, larger multicenter longitudinal studies are recommended to further explore this public health issue in the region and to inform more effective interventions.

## Introduction

Pertussis (whooping cough) is a respiratory infection caused by the bacterium *Bordetella pertussis*. Infants under one year old of age are particularly vulnerable to contracting whooping cough and may experience serious complications as a result [1]. All newborns are vulnerable to pertussis in the first two months of their lives. Pertussis is highly infectious and causes mortality in 1% of infected infants. According to the CDC, 24.1 million cases of whooping cough and about 160,700 deaths occur in children younger than five years worldwide annually [2]. Therefore, in 2012, the Advisory Committee on Immunization Practices recommended administering the pertussis vaccine in the third trimester of every pregnancy to provide temporary passive immunization to newborns and ensure immunity of mothers, consequently preventing pertussis-related illness and death among infants [3,4]. For women who did not receive the pertussis vaccine during pregnancy, it is still recommended to receive it postpartum to prevent the transmission of pertussis to newborns, as mothers were reported to be the primary source of infection to their newborns (mothers 39%, fathers 16%, and grandparents 5%) [3].

Although one of the most effective preventive measures against this disease is receiving the pertussis vaccine in the third trimester of pregnancy [4], the maternal pertussis vaccination (MPV) coverage rate remains suboptimal globally and locally [5-7]. This study assessed the level of awareness and knowledge of pertussis infection and vaccination among pregnant women and assessed the prevalence of MPV uptake among pregnant women in their third trimester and those who had delivered within 30 days (postpartum). It also highlighted the possible barriers to pertussis vaccination. This study aimed to provide a foundation for identifying the specific levels of intervention needed to address and improve the low uptake of MPV, ultimately contributing to better protection of infants against pertussis.

## Materials and methods

Study design

This was a cross-sectional study conducted at the outpatient obstetrics and gynecology clinic of King Abdullah Medical Complex (KAMC), a tertiary referral maternity hospital in Jeddah, Saudi Arabia. KAMC serves a substantial proportion of pregnant women across Jeddah, encompassing both low- and high-risk pregnancies. Data collection was carried out from September to December 2025.

Inclusion and exclusion criteria

Eligible participants included pregnant women in their third trimester (gestational age > 27 weeks) and women within 30 days postpartum, aged 18 years or older, of any nationality, who were Arabic-speaking. Women were excluded if they were non-Arabic-speaking, had a known allergy to the pertussis vaccine, were unable to complete the questionnaire, or were in the first or second trimester of pregnancy.

Ethical considerations

This study was approved by the Institutional Review Board of the Jeddah First Health Cluster at KAMC (approval number: A02247). All participants provided informed consent prior to enrollment.

Sampling and sample size

The minimum required sample size was calculated using the Raosoft sample size calculator [8], assuming an estimated target population of 2,800 patients per month, a 95% confidence level, a margin of error of 5%, and an expected response distribution of 50%. Based on these parameters, a minimum sample size of 338 participants was determined to be sufficient. Study participants were selected using simple random sampling.

Data collection

Data were collected using a validated, self-administered questionnaire adapted from MacDougall et al. [9], used under the Creative Commons Attribution-NonCommercial 3.0 license (CC BY-NC 3.0; creativecommons.org/licenses/by-nc/3.0). The change that was made was the removal of the attitude questions, as this was beyond the scope of the study. The instrument was translated into Arabic using a forward-backward translation procedure to ensure linguistic validity and cultural appropriateness.

The questionnaire comprised five sections. Section I captured sociodemographic characteristics, while Section II addressed pregnancy status and medical history. Section III assessed pertussis awareness, including prior knowledge of the disease, personal history of infection, perceived severity, and awareness of the pertussis vaccine. Section IV evaluated pertussis-specific knowledge, sources of health information, and informational needs related to vaccination decision-making. Section V examined MPV uptake, vaccine intention, willingness to receive the vaccine, and the role of the primary treating physician in recommending vaccination. Open-ended questions at the end of the questionnaire explored perceived barriers to MPV receipt.

Data collection was conducted by the primary researcher, who approached eligible women in the outpatient clinic and maternity ward, administered the questionnaire, and remained available to address any queries. Upon completion, participants received an educational pamphlet and video about pertussis and MPV to support awareness and informed decision-making.

Statistical analysis

All statistical analyses were performed using IBM SPSS Statistics for Windows, version 21.0 (released 2012; IBM Corp., Armonk, NY, USA). Continuous variables were summarized as means with SDs, and categorical variables were reported as frequencies and percentages. Pearson’s chi-square (χ²) test was used to examine associations between MPV uptake and sociodemographic characteristics, pertussis awareness and knowledge, maternal clinical history, prior MPV, sources of information, vaccine acceptance, and physician recommendation status.

For binary categorical variables, crude ORs with 95% CIs were derived from cross-tabulation risk estimates as part of Pearson’s chi-square analysis in SPSS. For variables with more than two categories (knowledge level and awareness level), crude ORs and 95% CIs were calculated using univariate logistic regression. Statistical significance was set at p ≤ 0.05.

## Results

Characteristics of participants

A total of 351 women participated in the study, of whom 46.7% were in their third trimester, and 53.3% were within 30 days postpartum. The majority were aged ≤35 years (75%), followed by those older than 35 years (24.5%). Most participants were Saudi nationals (85.4%) residing in Jeddah (92%), held a university-level education (67.5%), and were unemployed (83.5%). The mean number of pregnancies was 3.0 (SD 1.93; range 0-15), and the mean number of children was 2.0 (SD 1.57; range 0-7). Preexisting health conditions before and during pregnancy were reported by 30.2% and 37.6% of participants, respectively. A history of miscarriage, abortion, or stillbirth was reported by 61% of participants (Table 1).

**Table 1:** Characteristics of study participants and association with uptake of MPV ᵃ Age category ≤35 years includes participants aged 18-35 years; the minimum eligible age was 18 years per the inclusion criteria. ᵇ Four missing values were excluded from the analysis; percentages were calculated based on available data (n = 347). MPV, maternal pertussis vaccination

Characteristic	Category	N (%)
Pregnant in the third trimester	Yes	164 (46.7%)
	No	187 (53.3%)
Ageᵃ	≤35	265 (75%)
	>35	86 (24.5%)
Nationality	Saudi	300 (85.4%)
	Non-Saudi	51 (14.5%)
City of residence	Jeddah	323 (92%)
	Other cities	28 (8%)
Educational level	High school level or less	114 (32.5%)
	University level	237 (67.5%)
Employment status	Unemployed	293 (83.5%)
	Employed	58 (16.5%)
Number of childrenᵇ	≤1	137 (39 %)
	>1	210 (59.8%)
Health conditions before pregnancy	Yes	106 (30.2%)
	No	245 (69.8%)
Health conditions during pregnancy	Yes	132 (37.6%)
	No	219 (62.4%)
History of miscarriage, abortion, or stillbirth	Yes	215 (61%)
	No	137 (38.9%)
Knowledge level	Low	297 (84.6%)
	Moderate	36 (10.3%)
	High	9 (2.6%)
Awareness level	Low	137 (39%)
	Moderate	88 (25.1%)
	High	126 (35.9%)
Physician recommendation	Yes	6 (1.7%)
	No/unsure	345 (98.3%)
Intention to receive MPV (current pregnancy or postpartum)	Yes	109 (31.1%)
	No/unsure	242 (68.95%)

Prevalence and determinants of MPV uptake

Overall, MPV uptake was remarkably low, with only 10 of 351 participants (2.8%) having received the vaccine. However, 109 participants (31.1%) expressed an intention to receive MPV if it were offered free of charge. Given the very low MPV uptake observed in this study (n = 10, 2.8%), all subsequent association analyses should be interpreted with caution, as the small number of vaccinated participants limits the statistical reliability and generalizability of these findings. Univariate logistic regression identified four factors associated with MPV uptake (Table 2). Women with a high level of pertussis knowledge were significantly more likely to have received the vaccine compared to those with low knowledge (OR = 6.619; 95% CI: 1.814-24.151). Absence of or uncertainty regarding physician recommendation was associated with reduced uptake (OR = 0.971; 95% CI: 0.953-0.989). It should be noted, however, that while statistically significant, the ORs for Saudi nationality (OR = 1.034; 95% CI: 1.013-1.056) and city of residence, whereby women residing in Jeddah were less likely to be vaccinated than those from other cities (OR = 0.193; 95% CI: 0.047-0.789), should be interpreted with caution given the small number of outcome events (n = 10) and the near-null effect sizes observed for nationality.

**Table 2:** Factors associated with uptake of MPV ᵃ Four values were missing for the number of children; percentages were calculated based on available data (n = 347). ᵇ For knowledge level and awareness level, crude ORs and 95% CIs were calculated using univariate logistic regression; all other ORs and 95% CIs were derived from Pearson’s chi-square cross-tabulation risk estimates. ᶜ Denotes a statistically significant association (p ≤ 0.05).

Characteristic	Category	Crude OR	95% CI	χ²	p-Value
Pregnant in the third trimester	Yes	1.737	0.482-6.267	0.729	0.393
	No	1	Ref.	-	-
Age	≤35	1	Ref.	-	-
	>35	2.106	0.580-7.644	1.337	0.248
Nationality	Saudi	1.034	1.013-1.056	2.750	0.036ᶜ
	Non-Saudi	1	Ref.	-	-
City of residence	Jeddah	0.193	0.047-0.789	6.801	0.009ᶜ
	Other cities	1	Ref.	-	-
Educational level	High school level or less	4.52	0.566-36.114	2.372	0.124
	University level	1	Ref.	-	-
Employment status	Unemployed	0.786	0.163-3.800	0.090	0.764
	Employed	1	Ref.	-	-
Number of childrenᵃ	≤1	1.170	0.671-2.042	2.358	0.358
	>1	1	Ref.	-	-
Health conditions before pregnancy	Yes	2.376	0.673-8.387	1.589	0.368
	No	1	Ref.	-	-
Health conditions during pregnancy	Yes	1.685	0.479-5.934	2.028	0.321
	No	1	Ref.	-	-
History of miscarriage, abortion, or stillbirth	Yes	1.389	0.849-2.273	1.902	0.168
	No	1	Ref.	-	-
Knowledge levelᵇ	Low	1	Ref.	-	-
	Moderate	2.133	1.104-4.122	3.289	0.042ᶜ
	High	6.619	1.814-24.151	4.834	0.028ᶜ
Awareness levelᵇ	Low	0.87	0.538-1.407	0.698	0.404
	Moderate	1	Ref.	-	-
	High	1.387	0.491-3.914	0.714	0.258
Physician recommendation	Yes	1	Ref.	-	-
	No/unsure	0.971	0.953-0.989	4.012	0.029ᶜ
Intention to receive MPV (current pregnancy or postpartum)	Yes	1.498	0.414-5.421	0.385	0.535
	No/unsure	1	Ref.	-	-

Factors influencing intention to receive MPV

Among the 341 participants who had not received MPV, 109 (31.1%) expressed an intention to be vaccinated. Perceived severity of pertussis in adults was associated with vaccination intention, whereby participants who rated pertussis as a very high threat were more likely to intend vaccination (p = 0.001). Higher pertussis knowledge was also significantly associated with intention to receive MPV (p = 0.042). Additionally, prior vaccination with MPV was positively associated with intention to receive the vaccine in the current pregnancy or postpartum period (p = 0.032).

Pertussis awareness

Awareness of pertussis was generally low across the study population. Nearly half of the participants (49.6%) had never heard of pertussis prior to the study, and 93.2% reported no personal history of pertussis infection. Regarding perceived risk to infants, 49.6% of participants considered pertussis to pose a high threat to their newborns after delivery. Perceived severity among adults was notably lower, with only 39.6% rating pertussis as a high or moderately high threat to adults. Furthermore, 65.5% of participants were unaware that a vaccine exists to protect adults against pertussis infection (Table 3).

**Table 3:** Pertussis awareness questions and uptake of MPV among women ᵃ p-Values derived from Pearson’s chi-square test. MPV, maternal pertussis vaccination

Pertussis awareness questions/items	Uptake of MPV: Yes (n = 10)	Uptake of MPV: No (n = 341)	χ²	p-Valueᵃ
Before reading the information, have you ever heard of pertussis (whooping cough)?				
No/unsure (n = 210)	6 (2.9%)	204 (97.1%)	0.0001	0.992
Yes (n = 141)	4 (2.8%)	137 (97.2%)		
Have you ever had a pertussis infection?				
No/unsure (n = 346)	10 (2.9%)	336 (97.1%)	0.149	0.699
Yes (n = 5)	0 (0%)	5 (100%)		
How serious a threat do you think pertussis poses to a baby after delivery?				
High (n = 102)	2 (2.0%)	100 (98.0%)	4.454	0.216
Moderate (n = 72)	1 (1.4%)	71 (98.6%)		
Low (n = 39)	0 (0%)	39 (100%)		
Unsure (n = 138)	7 (5.1%)	131 (94.9%)		
How serious a threat do you think pertussis poses to adults?				
High (n = 9)	0 (0%)	9 (100%)	5.214	0.157
Moderate (n = 68)	0 (0%)	68 (100%)		
Low (n = 139)	3 (2.2%)	136 (97.8%)		
Unsure (n = 135)	7 (5.2%)	128 (94.8%)		
Before receiving the study information form, were you aware that there is a vaccine available that helps protect adults against pertussis infection?				
No/unsure (n = 285)	6 (2.1%)	279 (97.9%)	3.029	0.082
Yes (n = 66)	4 (6.1%)	62 (93.9%)		

Knowledge of pertussis

Overall, pertussis knowledge was poor, with high rates of “I don’t know” responses across all items. Knowledge of disease etiology was particularly limited, with 60.7% of participants unsure whether pertussis is caused by a virus. Less than half recognized pertussis as a highly contagious but vaccine-preventable disease (43.0%), and only 34.8% knew that pertussis can be fatal in newborns. Knowledge of transmission was similarly limited, with only 25.6% recognizing that pertussis can be transmitted to newborns immediately after birth. Only 31.9% were aware that maternal antibodies can be transferred to newborns through the placenta or breast milk, and an equal proportion knew that newborns primarily acquire pertussis from their mothers or close family members. Furthermore, only 19.7% correctly identified that the majority of pertussis-related deaths occur in infants under one year of age. Nearly four in five participants (78.9%) were unaware that infants under two months of age cannot receive the pertussis vaccine, and knowledge of vaccine safety during pregnancy, minor childhood illness, and breastfeeding was similarly deficient (Table 4).

**Table 4:** Pertussis knowledge questions and uptake of MPV ^*^ p-Value derived from Pearson’s chi-square test. MPV, maternal pertussis vaccination

Pertussis knowledge questions/items	Uptake of MPV: Yes (n = 10)	Uptake of MPV: No (n = 341)	χ²	p-Value
Pertussis is an acute respiratory infection that can cause a severe cough followed by a loud gasp or whoop sound before the next breath.				
True (n = 191)	4 (2%)	187 (98%)	1.071	0.585
False (n = 4)	0 (0%)	4 (100%)		
I don’t know (n = 156)	6 (4%)	150 (96%)		
Pertussis is caused by a virus.				
True (n = 123)	4 (3%)	119 (97%)	1.056	0.590
False (n = 15)	1 (7%)	14 (93%)		
I don’t know (n = 213)	5 (2%)	208 (98%)		
Pertussis is one of the most contagious diseases that can be prevented by vaccination.				
True (n = 151)	4 (3%)	147 (97%)	0.826	0.662
False (n = 15)	1 (7%)	14 (93%)		
I don’t know (n = 185)	5 (3%)	180 (97%)		
Pertussis in newborns can be deadly.				
True (n = 122)	3 (2%)	119 (98%)	1.377	0.502
False (n = 12)	1 (8%)	11 (92%)		
I don’t know (n = 217)	6 (3%)	211 (97%)		
Pertussis can be passed to the baby during pregnancy.				
True (n = 49)	4 (8%)	45 (92%)	5.903	0.052
False (n = 36)	1 (3%)	35 (97%)		
I don’t know (n = 266)	5 (2%)	261 (98%)		
Pertussis can be passed to newborns right after birth.				
True (n = 90)	4 (4%)	86 (96%)	1.572	0.456
False (n = 22)	0 (0%)	22 (100%)		
I don’t know (n = 239)	6 (3%)	233 (97%)		
Pregnant and breastfeeding women can pass antibodies that can protect against pertussis infection to their newborns through the placenta and breast milk.				
True (n = 112)	7 (6%)	105 (94%)	1.144	0.228
False (n = 18)	2 (11%)	16 (89%)		
I don’t know (n = 221)	1 (0.5%)	226 (99.5%)		
Newborns get pertussis mainly from their mother and other family members.				
True (n = 112)	3 (3%)	109 (97%)	0.502	0.778
False (n = 18)	1 (6%)	17 (94%)		
I don’t know (n = 221)	6 (3%)	215 (97%)		
Almost all pertussis deaths occur in children under one year of age.				
True (n = 69)	3 (4%)	66 (96%)	0.966	0.617
False (n = 24)	1 (4%)	23 (96%)		
I don’t know (n = 258)	6 (2%)	252 (98%)		
Newborns who have had pertussis are more likely to have learning or behavioral problems later in life.				
True (n = 39)	1 (3%)	38 (97%)	1.932	0.381
False (n = 53)	0 (0%)	53 (100%)		
I don’t know (n = 259)	9 (3%)	250 (97%)		
Adolescents and adults can get pertussis.				
True (n = 158)	3 (2%)	155 (98%)	1.881	0.390
False (n = 18)	0 (0%)	18 (100%)		
I don’t know (n = 175)	7 (4%)	168 (96%)		
Pertussis can occur in adults even if they have been fully immunized as infants.				
True (n = 62)	1 (2%)	61 (98%)	0.680	0.712
False (n = 47)	2 (4%)	45 (96%)		
I don’t know (n = 242)	7 (3%)	235 (97%)		
Infants and children are routinely immunized against pertussis.				
True (n = 66)	2 (3%)	64 (97%)	0.075	0.963
False (n = 28)	1 (4%)	27 (96%)		
I don’t know (n = 257)	7 (3%)	250 (97%)		
Adolescents and adults are routinely immunized against pertussis.				
True (n = 61)	3 (5%)	58 (95%)	1.225	0.542
False (n = 31)	1 (3%)	30 (97%)		
I don’t know (n = 259)	6 (2%)	253 (98%)		
Infants under two months of age cannot receive a vaccine against pertussis.				
True (n = 47)	1 (2%)	46 (98%)	0.161	0.923
False (n = 27)	1 (4%)	26 (96%)		
I don’t know (n = 277)	8 (3%)	269 (97%)		
It is safe to give the pertussis vaccine to a child whose mother is pregnant.				
True (n = 48)	0 (0%)	48 (100%)	4.750	0.093
False (n = 39)	3 (5%)	36 (95%)		
I don’t know (n = 264)	7 (3%)	257 (97%)		
It is safe to give the pertussis vaccine to a child who has a minor illness such as an ear infection.				
True (n = 82)	0 (0%)	82 (100%)	5.915	0.052*
False (n = 38)	3 (8%)	35 (92%)		
I don’t know (n = 230)	7 (3%)	223 (97%)		
Infants who are breastfeeding may receive all the recommended immunizations.				
True (n = 131)	5 (4%)	126 (96%)	1.379	0.502
False (n = 19)	1 (5%)	18 (95%)		
I don’t know (n = 201)	4 (2%)	197 (98%)		
Too many immunizations can overload or weaken the immune system.				
True (n = 56)	3 (5%)	53 (95%)	1.708	0.426
False (n = 64)	1 (2%)	63 (98%)		
I don’t know (n = 231)	6 (3%)	225 (97%)		

Barriers to vaccination uptake

Thematic analysis of participants’ responses indicated that negative or low uptake of the MPV was largely influenced by limited awareness and insufficient information. Many respondents were unaware of the vaccine, its role during pregnancy, or pertussis infection itself, and several noted that the vaccine had not been recommended by their healthcare provider (HCP). Concerns about vaccine safety were also frequently expressed, particularly fears of adverse reactions and allergies, with some preferring to delay vaccination until the postpartum period. In addition, low perceived susceptibility and severity of pertussis emerged as an important theme, as participants commonly viewed the infection as rare or not serious enough to warrant vaccination in the absence of illness or comorbidities. Moreover, participants reported that the internet and social media were their primary sources of information, followed by physicians. This highlights the importance of these channels as key players in addressing issues related to MPV uptake in the future.

Maternal concerns about vaccination 

Most of the women who participated in the study were concerned about vaccine safety and side effects. Nearly seven in ten respondents wanted more information about potential side effects they might experience (68.8%), followed by the safety of the vaccine for their baby (66.8%) and for themselves (65.6%). Concerns about risks associated with not receiving the vaccine were also common, particularly risks to the baby (64.2%) and to the mother (60.5%). Slightly fewer respondents indicated a need for information about possible side effects their baby might experience after vaccination (59.7%).

## Discussion

Since 2019, the Saudi Arabian Ministry of Health has recommended MPV at 27-36 weeks of gestation during every pregnancy, in alignment with CDC guidelines, with implementation extended across governmental, private, and other hospital sectors as well as all primary care clinics [10]. Notwithstanding this national directive, several studies conducted across Saudi Arabia have consistently documented low levels of awareness among both HCPs and pregnant women [11,12]. A 2023 study in the Kingdom revealed that only 47% of OB-GYNs acknowledged maternal immunization as their professional responsibility, and an equally low proportion were aware of the national recommendation to administer the pertussis vaccine during the third trimester of each pregnancy [11]. Furthermore, pregnant women in Taif demonstrated limited understanding of disease prognosis, transmission routes, and infection risk [11]. A separate study encompassing 231 women across multiple cities in Saudi Arabia found that the MPV was entirely unrecognized by the majority of participants [12].

The present study demonstrates persistently low awareness, limited knowledge, and low vaccination intention toward MPV among pregnant and recently postpartum women attending a tertiary care center in Jeddah. These findings are consistent with international evidence, including a study conducted in Thailand in which intention to receive pertussis vaccination during pregnancy was reported in only 45.5% of participants (95% CI: 40.5-50.4) [13].

Despite national recommendations endorsing routine MPV during every pregnancy, substantial informational and perceptual gaps persist, directly undermining maternal vaccination intentions and representing missed opportunities for infant protection. These results are in accordance with the broader body of evidence in the literature [9,11,12,14]. A randomized controlled trial conducted in Canada among 346 pregnant women similarly documented low knowledge of pertussis and its vaccine [9]. In the United States, MPV uptake during pregnancy was reported at 27.3%, a figure that underscores the global nature of this public health challenge [15].

The markedly low vaccine uptake observed in the present study is further corroborated by comparable findings internationally. In Italy, among 526 women, only 3.5% received an HCP recommendation to vaccinate against pertussis during pregnancy, 21% expressed willingness to do so, and a mere 1.7% actually received the vaccine, collectively reflecting very low uptake [14]. In contrast, a Canadian study reported that 89% of participants indicated a favorable intention toward vaccination when it was recommended by their HCP, highlighting the pivotal role of provider endorsement [9]. Regionally, a 2023 study in Taif found that only 15 of 401 women (3.7%) had received the pertussis vaccine during a previous pregnancy, further confirming the low uptake rates within the Saudi context [11].

The barriers contributing to low MPV uptake are multifactorial, including patient-level factors such as vaccine hesitancy, outright vaccine refusal, and low perceived susceptibility to pertussis [10,16]. In addition, system-level factors, such as knowledge and awareness deficits among pregnant women, compounded by HCPs’ limited confidence in initiating vaccination discussions, constitute additional significant barriers [10,16]. A robust body of literature underscores the critical role of HCP recommendations and proactive vaccine offerings in improving MPV rates [7,10,16]. Insufficient knowledge regarding vaccine efficacy, safety, and the diseases it prevents further impedes acceptance and uptake among pregnant women [10,16]. A 2023 Saudi Arabian study among 60 OB-GYNs identified anticipated patient refusal, inadequate familiarity with the disease and national guidelines, and concerns regarding vaccine availability and supply as principal barriers to pertussis vaccine administration [17]. Proposed strategies to enhance MPV uptake include continuing professional education for OB-GYNs and broad public awareness campaigns addressing the risks of pertussis infection, its potential complications, and the comparative benefits and risks of vaccination [17,18].

In the present study, MPV uptake was markedly low despite a generally favorable intention toward immunization during pregnancy, highlighting a substantial disconnect between willingness and actual vaccine receipt. Fewer than 3% of participants reported having received the MPV; however, nearly one-third expressed intention to vaccinate if the vaccine were offered free of charge, suggesting the presence of modifiable structural and informational barriers. Knowledge and awareness of pertussis and its vaccine were consistently limited across the study population, with considerable uncertainty regarding disease severity, transmission, and vaccine safety, particularly in the context of pregnancy and early infancy. Notably, higher knowledge scores were strongly and independently associated with both vaccine uptake and vaccination intention, underscoring the central role of informed decision-making in maternal immunization behavior. Consistent with existing literature, physician recommendations emerged as a key determinant of vaccine uptake, with the absence of or uncertainty surrounding HCP advice associated with significantly reduced vaccination rates. Qualitative findings further corroborated these patterns, identifying limited awareness, safety concerns, and low perceived susceptibility to pertussis as predominant barriers to uptake. Given that participants primarily relied on the internet and social media as sources of health information, followed by physicians, these findings collectively emphasize the imperative for strengthened provider-led counseling and the development of targeted, evidence-based educational interventions delivered through accessible and trusted channels.

Strengths and limitations

This study is strengthened by its adequate sample size, random sampling approach, and use of validated questionnaires adapted from prior research, along with conducting the study in a referral hospital that includes participants from all over Jeddah city and neighboring towns, which improves the external validity of this study. Also, inclusion of both pregnant and recently postpartum women allowed for broader insight into maternal perspectives and made these results timely. As for the limitations, it included the cross-sectional design, which precludes causal inference, and reliance on self-reported data. Additionally, open-ended responses were coded thematically by a single researcher, which may introduce subjectivity in the interpretation of qualitative data. Future studies are encouraged to employ multiple coders with inter-rater reliability assessment to strengthen the rigor of qualitative analysis. Furthermore, the very low MPV uptake (n = 10 vaccinated participants) limits the stability of the logistic regression model, as the number of outcome events is small relative to the number of predictors; findings from the univariate logistic regression analysis should therefore be interpreted with caution. However, there is consistency of the current study findings with national and international literature, which supports their validity and relevance.

## Conclusions

The observed MPV uptake of less than 3% in our study not only reflects low individual uptake but may also serve as an indicator of broader gaps in the implementation of preventive strategies at the healthcare system level. We recommend strengthening routine provider counseling during antenatal visits, improving vaccine accessibility, and implementing targeted educational interventions for pregnant women. Policymakers should consider revisiting the current implementation strategies of the national MPV program to ensure that recommendations are effectively translated into practice, ultimately reducing the burden of pertussis among vulnerable newborns and young infants.
